# Potential New Therapies “ROS-Based” in CLL: An Innovative Paradigm in the Induction of Tumor Cell Apoptosis

**DOI:** 10.3390/antiox13040475

**Published:** 2024-04-17

**Authors:** Raffaele Sciaccotta, Sebastiano Gangemi, Giuseppa Penna, Laura Giordano, Giovanni Pioggia, Alessandro Allegra

**Affiliations:** 1 Hematology Unit, Department of Human Pathology in Adulthood and Childhood “Gaetano Barresi”, University of Messina, Via Consolare Valeria, 98125 Messina, Italy; sciaccottaraffaele@gmail.com (R.S.); giuseppa.penna@polime.it (G.P.); laura.giordano@polime.it (L.G.); 2 Allergy and Clinical Immunology Unit, Department of Clinical and Experimental Medicine, University of Messina, Via Consolare Valeria, 98125 Messina, Italy; gangemis@unime.it; 3 Institute for Biomedical Research and Innovation (IRIB), National Research Council of Italy (CNR), 98164 Messina, Italy; giovanni.pioggia@irib.cnr.it

**Keywords:** reactive oxygen species, chronic lymphocytic leukemia, apoptosis, oxidative stress, lymphoproliferative diseases

## Abstract

Chronic lymphocytic leukemia, in spite of recent advancements, is still an incurable disease; the majority of patients eventually acquire resistance to treatment through relapses. In all subtypes of chronic lymphocytic leukemia, the disruption of normal B-cell homeostasis is thought to be mostly caused by the absence of apoptosis. Consequently, apoptosis induction is crucial to the management of this illness. Damaged biological components can accumulate as a result of the oxidation of intracellular lipids, proteins, and DNA by reactive oxygen species. It is possible that cancer cells are more susceptible to apoptosis because of their increased production of reactive oxygen species. An excess of reactive oxygen species can lead to oxidative stress, which can harm biological elements like DNA and trigger apoptotic pathways that cause planned cell death. In order to upset the balance of oxidative stress in cells, recent therapeutic treatments in chronic lymphocytic leukemia have focused on either producing reactive oxygen species or inhibiting it. Examples include targets created in the field of nanomedicine, natural extracts and nutraceuticals, tailored therapy using biomarkers, and metabolic targets. Current developments in the complex connection between apoptosis, particularly ferroptosis and its involvement in epigenomics and alterations, have created a new paradigm.

## 1. Introduction

### 1.1. General Considerations on Chronic Lymphocytic Leukemia

An essential characteristic of chronic lymphocytic leukemia (CLL), a kind of hematological cancer, is the presence of mature monoclonal B-lymphocytes that have been accumulating for an extended period of time in the bone marrow, lymph nodules, and peripheral circulation [1]. The categorization of lymphoid neoplasms by the World Health Organization (WHO) recognizes CLL as a slow-growing B-cell lymphoma [2]. With an incidence of 4.2/100,000/year, CLL is the most prevalent kind of leukemia in the Western world. The frequency rises to around 30 per 100,000 each year in those over 80 years old. The median age of diagnosis is 72 years. Approximately 10 percent of individuals diagnosed with CLL are younger than 55 years old. Individuals with a family history of CLL have a much higher chance of developing the disease due to an inherent genetic predisposition, with family members having a sixfold to ninefold greater risk [3]. In order to diagnose CLL, it is necessary to have documented evidence of the existence of 5 × 10^9^/L B cells in the peripheral blood over a period of at least three months. The leukemia cells seen in a blood sample are tiny, mature-looking lymphocytes with little cytoplasm, a compact nucleus, no visible nucleoli, and somewhat clumped chromatin. During the process of slide preparation, CLL cells have a tendency to break, and the debris that results from this process may be easily spotted and referred to as Gumprecht shadows or basket cells [4]. Flow cytometry analysis is necessary for diagnostic confirmation because it reveals the specific phenotype of the clonal population, which is defined by the co-expression of the B-cell surface antigens CD19 and CD20 with CD5, CD23, CD43, and CD200. Surface levels of CD20, surface immunoglobulin (Ig), and CD79b are typically lower than those present on healthy B cells. Each clone of leukemia cells is limited to expressing either kappa or lambda Ig light chains or shows no detectable expression of either [5]. Asymptomatic people who are discovered to have leukocytosis with lymphocytosis on a routine blood test are one example of the clinical manifestations of CLL. Other patients may have painless enlargement of superficial lymph nodes as a symptom of CLL [4]. In general, 5–10% of patients suffer from typical B symptoms of lymphoma. These symptoms include unintentional weight loss that is greater than 10% of their body weight within the preceding six months, fevers that last for two weeks without any signs of infection, drenching night sweats, and weakness. Sometimes, individuals are identified when they show symptoms associated to autoimmune conditions such autoimmune hemolytic anemia or immune-mediated thrombocytopenia [6]. Clonal B cells are likely developed at the hematopoietic stem cell (HSC) stage, indicating that the initial event leading to leukemia in CLL may involve multipotent, self-regenerating HSCs. The step-by-step mechanism of leukemogenic transition is becoming widely recognized [7]. Around 80% of patients with CLL have one of four common chromosomal changes: deletion in chromosome 13q14.3 (del[13q]), deletion in chromosome 11q, deletion in chromosome 17p, or trisomy 12 [8]. A percentage ranging from 5% to 8% of chemotherapy-naïve individuals are discovered to have deletions of the short arm of chromosome 17, frequently referred to as del(17p). Deletions commonly include band 17p13, which is the location of the important cancer suppressor gene TP53. Patients with CLL who have a del(17p) exhibit significant resistance to chemotherapies that damage genetic material [9]. The “mutational architecture” and broad genetic variability of primary CLL samples have been uncovered by large-scale genome-wide whole-exome and whole-genome sequencing investigations conducted during the past 10 years on these samples [10]. The combination of somatic mutations and clinical data allowed for the discovery of genetic drivers and enhanced the prediction of outcomes for patients with CLL [11]. Some of the most critical cellular pathways contain genes that have been identified to be mutated. These include genes involved in metabolism, RNA splicing, DNA damage, MAPK-ERK, cell cycle, NF-KB signaling, chromatin modification, and B-cell receptor signaling (Table 1) [12]. One of the most important steps in the formation of tumors is the inactivation of p53, which is also known as TP53 and TRP53. This process is connected to the disruption of the cell cycle, instability in the genome, and the ability to survive chemotherapy [13]. According to a number of research studies, mutations in TP53 are detected in 4–37% of patients who have CLL, and they have been linked to a very bad prognosis [14]. The majority of patients with verified del(17p) have mutations in the remaining TP53 allele, with a frequency exceeding 80%. TP53 mutations are less common in instances lacking del(17p), but nevertheless have a significant negative impact on chemotherapeutic response and overall survival [15]. Additionally, dysregulation of the “apoptotic machinery” is a crucial factor in the development of CLL. When CLL cells are stimulated in a laboratory setting, their proliferation is induced by engaging the BCR, TLR, CD40, or cytokines receptors [16,17,18]. Researchers have shown that the enhanced ability of activated CLL cells to resist apoptosis is reliant on the activation of alternative BCL2 family members, such as BCL2A1 and BCL-XL, through the use of CD40 signaling and IL4 stimulation [19,20]. The excessive expression of Bcl-2 leads to the formation of malignancies, namely in CLL and follicular lymphoma. Additionally, it induces aberrant cell viability and imparts resistance to therapeutic interventions [21,22]. BH3-only proteins can initiate apoptosis in reaction to significant stresses, such as genetic harm [21]. As a result, increased levels of Bcl-2 neutralize the actions of BH3-only proteins, preventing apoptosis [23]. The upregulation of Mcl-1 and XIAP genes in CLL cells leads to the development of an antiapoptotic phenotype [24,25]. Moreover, the epigenome of chronic lymphocytic leukemia has emerged as an additional characteristic that defines the illness [26]. Increasing populations of CLL cells evolve by random alterations in methylation of DNA, known as epimutations [27]. The presence of genetic heterogeneity serves as a source of fuel for clonal expansion and is associated with the course of illness as well as a bad prognosis [28]. Further research has revealed that genetic diversity is affected by the cell type where some mutations are mostly found, such as in the immunoglobulin heavy-chain variable region gene (IGHV) unmutated CLL (U-CLL) (*U1snRNA*, *NOTCH1*, *POT1*) [29,30]. Two staging methods, Rai and Binet, are generally approved for use in both patient care and clinical research [31,32]. The first Rai classification was revised to decrease the quantity of prognostic groupings from five to three [33]. Consequently, both methods currently categorize three main categories with clearly defined clinical results. Each of these two staging methods is straightforward, low-cost, and can be utilized by medical professionals all across the world in a straightforward and uniform manner. Both depend exclusively on physical examinations and normal laboratory testing and do not need imaging investigations [31,32]. Typically, in medical practice, individuals with early stages illness (Rai 0, Binet A) who do not show any symptoms should be observed without treatment, unless there is evidence of disease development or symptoms connected to the condition. Numerous investigations have demonstrated that there is no survival advantage associated with the treatment of individuals who are in the early stages of illness [34,35]. While patients diagnosed with intermediate-stage (I and II) and high-risk (III and IV) disease, as determined by the modified Rai classification or Binet stage B or C, typically experience positive outcomes from starting treatment, certain patients (specifically those with Rai intermediate-risk or Binet stage B) can be observed without therapy until they show signs of gradually or symptomatic disease, referred to as ‘active disease’. In order to begin treatment, it is necessary to provide clear documentation of the “active disease” [1] (Table 2). 

**Figure 1:**
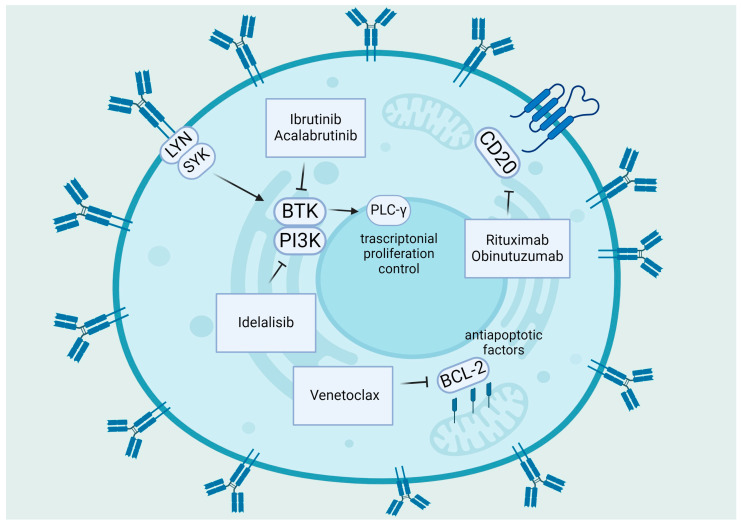
Summary of target therapies in CLL: The primary pathogenic pathways of CLL and the medicines that target BTK, PI3K, and Bcl-2. BCR signaling is triggered by self-binding or antigen recognition; Lyn encourages spleen tyrosine kinase (Syk) activation. After that, Syk initiates the assembly of a multi-component “signalosome” that consists of Btk (inhibited by ibrutinib, acalabrutinib), PI3K (inhibited by idelalisib), and PLCγ2. Anti-apoptotic molecules such as Bcl-2 (inhibited by venetoclax), Bcl-XL, and Mcl-1 are upregulated in CLL, whereas pro-apoptotic molecules Bax and Bak are sequestered, and the intrinsic apoptosis pathway is inhibited [37]. “Created with BioRender.com”.

Various therapeutic methods have been developed for front-line therapy, including regular treatment with Bruton tyrosine kinase inhibitors (BTKis) like ibrutinib/acalabrutinib until disease progression, or time-limited therapy with recently granted approval combination of the venetoclax and obinutuzumab or chemotherapy (ChT) backbone and CD20 antibodies [36]. Figure 1 provides an overview of the primary biological objectives of chemotherapy-free treatments in CLL [37].

### 1.2. ROS, Cancer, and Apoptosis

**Figure 2:**
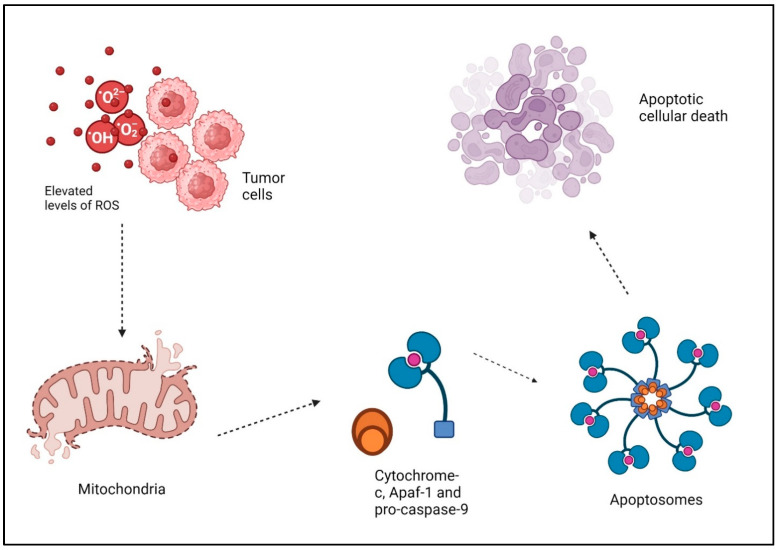
Elevated levels of ROS and tumor cells apoptosis: ROS disrupt the membrane of the mitochondria and activate the mitochondrial electron transport chain (ETC), leading to the release of cytochrome c and the opening of the permeability transition pore (PTP). Cytochrome-c, together with Apaf-1 and pro-caspase-9, produces “apoptosomes” in the cytosol. These apoptosomes trigger caspase-9, which then initiates executioner caspases like caspase-3 or 7. This ultimately leads to the breakdown of proteins and the occurrence of apoptotic cellular death. “Created with BioRender.com”.

Reactive oxygen species (ROS) are oxygen-based free radicals, including superoxide and other molecules like hydrogen peroxide. ROS are generated by a variety of external and internal sources. There are a number of endogenous enzymes that are responsible for the production of free radicals, the most significant of which are the electron transport chain (ETC) and the transmembrane NADPH oxidases (NOXs) on the inner mitochondrial membrane [38]. Oxidants can also be formed by enzymes, such as xanthine oxidase, cyclooxygenases, nitric oxide synthase, cytochrome P450 enzymes, and lipoxygenases. Peroxisomes and the endoplasmic reticulum (ER) are also capable of producing oxidants themselves [39]. ROS serve two roles in cellular metabolism. More precisely, at low to moderate concentrations, they function as signal messengers that stimulate the growth of new blood vessels, invasion of surrounding tissues, migration of cells, and cell division [40]. These species exhibit high reactivity and possess the capability to engage with complex biological molecules, causing modifications to the structure and function of proteins, ultimately resulting in the oxidative breakdown of [41]. Cancerous cells produce more ROS than normal cells because of their overactive metabolism, signaling pathways, peroxisomal activity, dysfunctional mitochondria, activation of oncogenes, and increased biological activity of oxidases, cyclooxygenases, lipoxygenases, and thymidine phosphorylases [42]. The damage to cellular DNA caused by ROS is mostly attributed to the oxidative stress-induced degradation of pyrimidine and purine bases, single-strand breaks, and oxidation of proteins thiols and lipids [43]. Therefore, elevated levels of ROS can trigger signaling pathways that stimulate apoptosis in malignant cells [44]. DNA damage buildup hampers the replication and repair of cancer cells’ DNA, leading to cell cycle halt and eventual cell demise. ROS, while directly impacting cancer cells, additionally has a critical role in regulating the immune system’s defense against tumors. Among these ROS, hydrogen peroxide (H_2_O_2_) is particularly noteworthy due to its direct and potent capacity to induce apoptosis. ROS disrupt the membrane of the mitochondria and activate the mitochondrial electron transport chain (ETC), leading to the release of cytochrome c and the opening of the permeability transition pore (PTP) [45]. Cytochrome-c, together with Apaf-1 and pro-caspase-9, produces “apoptosomes” in the cytosol. These apoptosomes trigger caspase-9, which then initiates executioner caspases like caspase-3 or 7. This ultimately leads to the breakdown of proteins and the occurrence of apoptotic cellular death (Figure 2) [46]. The extrinsic pathway is initiated when death-inducing ligands bind to certain receptors, including Fas ligands and TNF-related apoptosis-inducing ligands (TRAIL-R1/2), which then recruit pro-caspases and adaptor elements. Consequently, the formation of the death-inducing signaling complex (DISC) occurs, leading to the activation of effector caspases and initiation of apoptosis [47]. In addition, Caspase-8 and Caspase-10 break Bid, resulting in the formation of truncated Bid (tBid). tBid then moves to the mitochondria, where it hinders the anti-apoptotic function of BcL-2 and BcL-xL while also activating Bax and Bak [47]. Moreover, the production of oxidative stress inside mitochondria is essential for the initiation of the p53-induced intrinsic apoptotic signaling pathway [48]. Indeed, ROS induce the secretion of chemokines and cytokines, which recruit immune cells to the tumor microenvironment (TME) [49]. Moreover, ROS augment the ability of dendritic cells to deliver antigens, hence enabling the stimulation of T cells and fostering a comprehensive immune defense against tumors [50]. Additionally, the effectiveness of various cancer therapies, such as radiation therapy and certain chemotherapy drugs, can be improved by ROS [46]. Radiation relies on the generation of ROS to cause DNA damage and eliminate cancer cells. Utilizing ROS-generating treatments with traditional methods can result in synergistic benefits, improving the effectiveness against tumors [51,52]. Cancer stem cells are thought to have a role in the beginning, advancement, and reappearance of tumors. These cells frequently demonstrate resistance to traditional treatments, presenting a difficulty for effective therapy. ROS have been discovered to specifically attack cancer stem cells by causing oxidative stress. Targeting these cells specifically, medicines based on ROS show potential in preventing tumor recurrence [53,54,55]. 

### 1.3. Cancer Therapy and ROS

Within the realm of cancer chemotherapy, therapeutic selectivity is among the most crucial factors to take into account. Developing therapeutic ways to selectively eliminate cancerous cells with little impact on healthy cells relies on comprehending the molecular distinctions between cancerous and ordinary cells. While anticancer drugs have shown effective against several types of malignancies, numerous studies have established their toxicity [56,57]. Changes in the cellular ROS levels are known to be significant in causing apoptotic cell death [58,59]. However, the possibility of using modulation of oxidative stress to induce apoptosis and neoplastic cell death must address numerous issues related to efficacy and safety. In cancer cells, ROS are generated and metabolized differently than in normal cells, but many effects of oxidative stress are known to be tumor and cell specific. Different results can therefore be expected in different types of neoplasia. A second problem is the approach itself. To manipulate ROS levels therapeutically, one can modify ROS metabolic enzymes like SOD or utilize substances that boost ROS generation either directly or indirectly. As an example, doxorubicin, bleomycin, and arsenic trioxide are all examples of anticancer drugs that kill cancer cells through methods that include the formation of ROS. These compounds are now utilized in cancer treatment, and combining them with novel anticancer drugs might be a strategic approach to boost therapeutic effectiveness [60,61,62,63,64].

#### 1.3.1. Role of ROS and Other Mechanisms of Cell Death

##### Necroptosis

An alternative to apoptosis that occurs when death domain receptors are activated is necroptosis, a form of programmed cell death that causes inflammation [65]. The phenomenon displays morphological characteristics associated with both apoptosis and necrosis. Necroptosis is triggered by the interaction between death receptors and their specific ligands, as well as by genotoxic stressors or some anti-tumor medications such as etoposide [66]. The relationship between TNFR superfamily members and TNF-α, the principal mediator of cell death including apoptosis and necroptosis, triggers a signaling pathway that has common components with apoptosis and maintaining cells [67]. Out of all the death receptors, TNF-α is the most extensively studied due to its involvement in the process of necroptosis [68]. It necessitates the presence of protein RIPK3 (receptor-interacting protein kinase 1) and its substrate MLKL, which are essential components of this pathway [69]. Several processes, including carcinogenesis, tumor growth, cancer death, tumor spread, and cancer immunological response, are influenced by the necroptotic signaling system [70]. 

Necroptosis is crucial in regulating human immunity. An earlier study performed by Aaes et al. shown that cells undergoing necroptosis can emit damage-associated molecular patterns (DAMPs), such as HMGB1 and ATP, as well as chemokines like CXCL1. This led to the development of dendritic cells (DCs) generated from bone marrow, the presentation of CD8+ T lymphocytes, and the production of IFN-γ. Consequently, the BALB/c animals with a fully functioning immune system that were injected with necroptotic DD_RIPK3 cells exhibited potent anti-tumor reactions [71] A separate study showed that when cancer cells die, they stimulate the release of IL-12 by DCs. This process creates a positive feedback loop that promotes necroptosis and the removal of cancer cells [72]. Prior research has indicated a connection between necroptosis and reactive oxygen species (ROS) in situations involving abnormal physiological circumstances. One example is curcumol, which is obtained from the roots of Rhizoma curcumae. This compound has the ability to activate the JNK signaling pathway and enhance the production of ROS in hepatic stellate cells through a process called necroptosis that is reliant on RIP1/RIP3 [73]. In another investigation carried out by Zhang, it was demonstrated that the modification of cysteine (C) residues at positions 257, 268, and 586 by ROS can lead to the creation of an intramolecular disulfide bond. This bond facilitates the autophosphorylation of RIP1 at serine (S) 161 and subsequently attracts RIP3 to form the necrosome. Consequently, this suggests that mitochondrial ROS produced by TNF act as a catalyst for necroptosis [74]. Additionally, several studies have demonstrated that the generation of ROS can induce necroptosis, either through ceramide formation or as a result of increased energy metabolism mediated by NOXs or the mitochondrial ETC [69]. Therefore, the aforementioned data indicate a connection between ROS and necroptosis and that they have a positive correlation. Recent findings suggest a strong association between ROS and necroptosis, a kind of cell death. In addition, controlling ROS levels is seen as a potential treatment for cancer [75]. Therefore, targeting cancer cells for elimination through necroptosis and the generation of ROS appears to be a promising approach [76]. 

##### Ferroptosis

The Stockwell Lab undertook a high-throughput screen in 2001 to uncover new small molecule anti-cancer medicines. This led to the identification of chemicals that trigger a distinct type of cell death, published in 2003 [77]. The results of the antagonist screening showed that this particular form of cell death was prevented by a number of iron chelators and lipophilic radical-trapping antioxidants (RTAs) [78]. The need for iron led to the creation of the word “ferroptosis”. Studies have found that ferroptotic cell death may be triggered by inhibiting two specific cellular elements: system xc−cystine/glutamate antiporter and GPX4 [79]. To support what has been said, the detailed examination of the initial conditional Gpx4 knockout mouse model by the Conrad group revealed that the absence of Gpx4 leads to lipid-peroxidation-induced, non-apoptotic cell death in murine embryonic fibroblasts, as well as degeneration of neurons in the hippocampus and cortical areas of the brain. This process involves an antiporter protein and GPX4, which are blocked by the compounds erastin and RSL3, respectively [80]. Ursini and colleagues identified GPX4, a selenoprotein, as the main enzyme that reduces phospholipid hydroperoxides (PLOOHs) in mammalian cells by biochemical techniques [81]. The reason ferroptosis relies on iron may be understood by considering the crucial role of cellular metabolism, particularly phospholipid peroxidation, in this kind of death. Lipoxygenases and cytochrome P450 oxidoreductase are metabolic enzymes involved in phospholipid peroxidation that rely on iron for catalysis. Iron is also crucial for several metabolic enzymes responsible for producing cellular ROS. Secondly, the non-enzymatic Fenton chain reaction, which is dependent on iron, is probably a crucial component of ferroptosis. When GPX4 is blocked, PLOOHs are able to remain for a longer period of time, which triggers the Fenton reaction to quickly multiply PLOOHs, which is the characteristic feature of ferroptosis [82]. Efforts are now being made to create cancer treatments that are based on the stimulation of ferroptosis. Various nanoparticle-based methods have been explored to transport iron, peroxides, and other harmful substances to eliminate tumor cells [83]. The existence of numerous enzymes regulating ferroptosis allows for the creation of specific delivery methods [84]. A recently discovered ROS-associated pathway of p53’s tumor regression has shown an intriguing process that induces ferroptosis, which is reliant on the presence of iron within cells, by elevating concentrations of ROS [85]. To support this, the experiment conducted by Yang et al. utilized several chemicals known as ferroptosis-inducing substances (FINs). It was shown that each FIN inhibited the enzyme GPX4, either by direct or indirect means, by depleting the antioxidant glutathione (GSH). Their findings established GPX4 as the primary regulator of ferroptosis [79,86].

## 2. CLL and Oxidative Stress

### 2.1. CLL and ROS—General Considerations

CLL cells have inherently elevated amounts of ROS and experience oxidative stress as a result of an unbalanced redox state, in contrast to regular lymphocytes [87]. The oxidative degradation of amino acids containing sulfur through ROS regulates the function and role of proteins, including phosphatases and transcription factors (e.g., NF-κB, p53, hypoxia-inducible factor-1α, and nuclear factor erythroid 2-related factor 2), in modifying cellular development and survival [88]. Increased amounts of ROS also make CLL cells more susceptible to substances that further enhance ROS and oxidative stress [89]. Nrf2 triggers the activation of genes that participate in the cellular response to oxidative stress. These include genes such as heme oxygenase-1 (HMOX-1) and glutamate cysteine ligase modifier (GCLM), that are responsible for the production of glutathione (GSH) [90]. In CLL lymphocytes, excessive concentrations of ROS can surpass antioxidant defenses and cause the oxidation of proteins, resulting in the buildup of potentially harmful, misfolded, and polyubiquitylated (poly-Ub) proteins within cells [91]. The build-up of this substance activates a heat shock and proteo-toxic stress response that is mediated by HDAC6, serving as an adaptive and protective mechanism [92,93]. In this process, HDAC6 attaches to the poly-Ub-misfolded proteins and transports them to a safe aggresome. At the same time, this causes the breakup of the p97/HDAC6/hsp90/HSFl complex, leading to the activation of HSF1 and HSPs gene expression [94,95]. The detachment of HDAC6 from this compound also leads to excessive acetylation and suppression of the chaperone activity of hsp90, resulting in the reduction of CLL-related, growth-promoting, and survival-promoting hsp90 [96] client proteins, including ZAP70, c-RAF, AKT, and finally HDAC6 itself [97,98,99,100]. Furthermore, elevated amounts of NO in CLL cells result in heightened production of newly developed mitochondria [101], leading to an increase in oxidative phosphorylation when exposed to oxygen [102]. The microenvironment reduces oxidative stress, which may be simulated in a laboratory setting by coculturing CLL cells to enhance the thiol content [103]. 

#### 2.1.1. Microenvironment and Oxidative Stress in CLL

**Figure 3:**
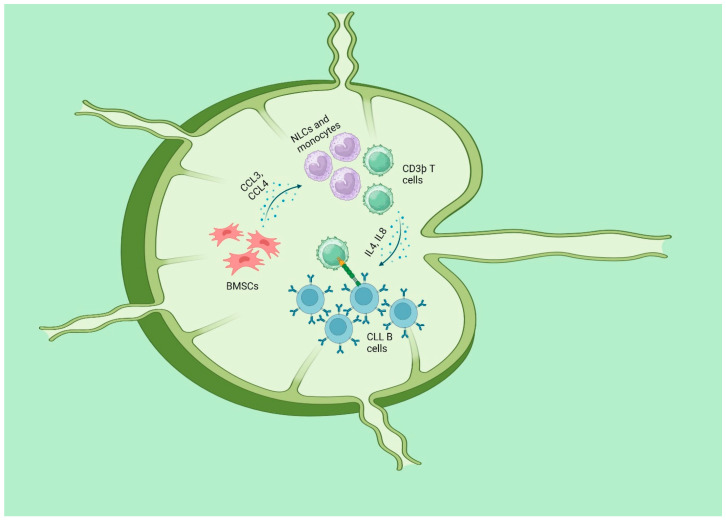
CLL and microenvironment: BMSCs and NLC, present in lymph nodes, emit potent chemotactic signals that facilitate the attraction and retention of circulating CLL cells inside the tissues. Stromal cells release chemokines such CCL3 and CCL4 to recruit more CD3þ T cells and monocytes, so creating a more favorable milieu. The recruited T cells are mostly CD4þ/CD154(CD40L)þ and have a significant impact on the stimulation of CLL cells, partly through the involvement of the TNF superfamily member CD40. CD40 ligation, when combined with Tcell–derived cytokines like IL4 and IL8, increases the survival, growth, and resistance to the standard immunochemotherapy of CLL. “Created with BioRender.com”.

CLL is identified by the growth of a single kind of fully developed B cells expressing CD5 and CD23 in the blood, bone marrow (BM), and secondary lymphatic organs (SLO, such as the lymph nodes and spleen). CLL cells retain certain functional traits of normal B lymphocytes, such as essential signaling pathways, including the B-cell receptor (BCR) and its following signaling cascade. These pathways normally regulate B-cell selection and proliferation, which are vital to adaptive immune responses [104]. Both the survival and multiplication of CLL cells are dependent on signals that come from the microenvironment in SLO. The SLO was shown to be the major location of CLL cell proliferation, as revealed by direct in vivo studies [105,106]. The CLL clone consists of a small portion of CLL cells that are actively proliferating, mostly seen in SLO, and a larger portion of B cells that are in a resting state [107]. The CLL cells derived from these different areas have distinct features in terms of their morphology, gene expression, and immunophenotype. The proliferative region of SLO generates specific formations called “proliferation centers (PC)”, commonly known as “pseudofollicles”. These features are frequently detected in the histology of CLLs [108]. When CLL cells are removed from their surrounding environment, they undergo programmed cell death unless they are cultured with either bone marrow stromal cells (BMSC) [109,110] or monocyte-derived nurse-like cells (NLC) [111]. BMSCs and NLC, present in lymph nodes, emit potent chemotactic signals that facilitate the attraction and retention of circulating CLL cells inside the tissues [111]. After taking up residence, CLL cells establish intricate two-way connections with stromal cells. These stromal cells release chemokines such CCL3 and CCL4 to recruit more CD3þ T cells and monocytes, creating a more favorable milieu [112,113]. The recruited T cells are mostly CD4þ/CD154(CD40L)þ and have a significant impact on the stimulation of CLL cells, partly through the involvement of the TNF superfamily member CD40 [113]. CD40 ligation, when combined with T-cell-derived cytokines like IL4 and IL8, increases the survival, growth, and resistance to the standard immunochemotherapy of CLL (Figure 3) [114,115,116,117,118]. A comparative analysis was conducted to examine the transcriptomes of primary CLL cells that were either cultivated alone or mixed with BMSCs known for their protective properties. The findings revealed that CLL cells without protection have significant disruption in the processes of oxidative phosphorylation, mitochondrial activity, and hypoxia signaling. The climax of these changes takes place within a time frame of 6–8 h, just before the start of apoptosis. Some CLL patients had a gene expression pattern similar to that of CLL cells grown in a culture together and experienced considerably worse outcomes in terms of both progression-free survival and overall survival. In order to discover medications that inhibit the assistance provided by BMSCs, the investigators analyzed the transcriptome alterations associated with this process to the Connectivity Map database. A correlation was identified between the transcriptome profiles of the cardiac glycoside ouabain and the ipecac alkaloids emetine and cephaeline. These substances, which functioned by suppressing HIF-1α and upsetting intracellular redox homeostasis, were highly effective against protected primary CLL cells (relative IC50s of 287, 190, and 35 nM, respectively). Pathways that control the balance of oxidative and reductive reactions are potential targets for therapeutic intervention in CLL cells since they play a crucial role in maintaining a favorable microenvironment for these cells [119]. Additionally, BMSCs exert an influence on the metabolic processes of nascent CLL cells via enhancing glycolysis [120]. Furthermore, regarding active CLL cells, stimuli from the microenvironment might potentially induce signals that alter the balance away from the apoptotic reaction of p53 and towards a pro-survival reaction from p53. The determination of the most crucial components in influencing this conclusion is still pending. The precise mechanisms that govern cell fate following p53 activation remain incompletely elucidated. The choice to undergo apoptosis, arrest, or any of the normal p53 responses is governed by a combination of characteristics that regulate p53 activity. The mechanisms influencing this phenomenon encompass the levels of proteins and ROS [121], the presence of oxygen [122], and a specific pattern of posttranslational modifications of p53 [123]. The p53 protein is upregulated by a factor of 30–40 after transcription in CLL B cells under circumstances that imitate those seen in lymph nodes. The phosphorylation of induced p53 enables it to perform certain, but not all, of its transcriptional activation tasks. Furthermore, Samuel et al. demonstrated that the upregulation of p53 protein is contingent upon a simultaneous and significant elevation in intracellular ROS and the subsequent oxidative DNA damage. The authors suggested a theoretical framework in which the baseline levels of active p53 are naturally increased in CLL cells that have been stimulated, as a result of ongoing DNA damage caused by oxidative stress. These cells would exhibit greater resistance to p53 due to the occurrence of antiapoptotic and pro-survival messages, such as those produced by BCL2-related proteins and p53 itself [124]. Through activation of the checkpoint kinase Chk1, increased levels of ROS within the cell can result in the cell cycle being stopped in the G2-M phase. The activation of the p53 homologue p73 and the subsequent transcription of NOXA are both triggered as a result of this. The DNA damage response defects that are present in cancer cells that lack functioning p53 may be overcome with the assistance of this mechanism [125].

#### 2.1.2. ATM Gene and Oxidative Stress

**Figure 4:**
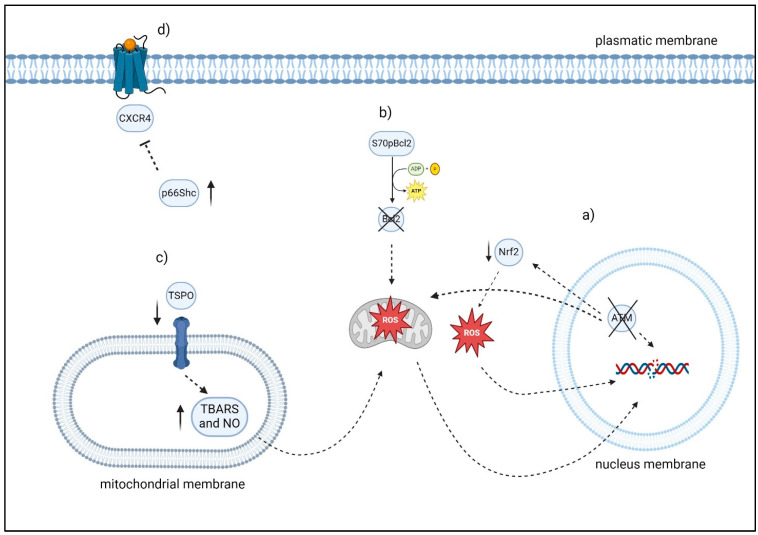
Overview of factors modulating oxidative stress and apoptosis in CLL cells: (**a**) ATM may be able to manage oxidative stress by exerting an influence on NRF2. Moreover, cells lacking ATM in CLL exhibited decreased antioxidant levels and increased mitochondrial ROS. (**b**) S70pBcl2 reduces the binding of Bcl-2 to the mitochondrial complex-IV subunit-5A, leading to an impact on the function of mitochondrial complex-IV, respiration, and the production of ROS. (**c**) In the De Rosa et al. investigation, the lymphocytes of 30 consecutive patients with CLL exhibited elevated levels of TSPO expression, decreased levels of TBARS, NO, which are two indicators of oxidative stress and caspase-3 activity. After six months of therapy, the ratio of TSPO to mitochondria in 24 out of 30 patients with CLL approximated that of healthy individuals. Significantly, the six patients who exhibited resistance to therapy also demonstrated elevated TSPO levels, reduced caspase 3 activity, and decreased TBARS levels. (**d**) The level of p66Shc expression was observed to have an inverse correlation with chemokine receptor expression and the degree of organ infiltration. “Created with BioRender”.

The ATM gene, discovered in 1995 [126], is of considerable size, consisting of 66 exons and having an open reading frame spanning 9168 nucleotides. The ATM gene product, ATM, is a protein kinase consisting of 3050 amino acids and is classified as a member of the phosphoinositide 3-kinase-related protein kinase superfamily. The ATM protein is mostly localized in the nucleus; however, it has also been shown in the cytoplasm where it is linked with peroxisomes [127]. ATM, a versatile protein kinase, has a crucial function in controlling cell cycle, repairing DNA damage, and determining cell survival and death. It achieves this by phosphorylating various substrates after undergoing autophosphorylation [128]. *ATM* gene mutations are present in 14–16% of CLL. The mutations are distributed throughout a broad coding region consisting of 64 exons. The depletion of ATM function often happens as a result of the combined impact of 11q deletion (monoallelic ATM loss) and an ATM mutation, biallelic ATM mutations, or, less commonly, owing to the presence of a single mutation that is capable of having a dominant-negative influence on whatever *ATM* alleles are still present. On the other hand, CLL cancers that have both ATM alleles inactivated exhibit lower DNA damage-induced apoptotic responses in vitro and faster clonal proliferation in vivo. This leads to a loss in overall and treatment-free survival when compared to CLL tumors that only have 11q deletion [129]. ATM plays a role in the process of preserving redox equilibrium. The oxidative stress that results from a disruption of the redox equilibrium can be brought on by either an increase in the quantities of ROS or a reduction in the antioxidant capacity of the organism. According to research conducted on Ataxia Telangiectasia (A-T), a human radiation sensitivity disorder in which both *ATM* alleles are disabled, the most persuasive evidence indicating the function of ATM in maintaining redox equilibrium is gained from this condition. The cells of these people exhibit elevated levels of oxidative stress as a consequence of the presence of high levels of ROS, an increased ratio of oxidized to reduced glutathione (GSSG/GSH), a diminished capacity to eliminate ROS, and impaired mitochondrial function [130]. Additionally, oxidative stress is the direct activator of ATM [131], and it is able to exhibit antioxidant action through the control of the pentose-phosphate pathway [132]. According to recent discoveries, ATM may be able to manage oxidative stress by exerting an influence on NRF2 (Figure 4). It was shown that the expression of *Nrf2* target genes decreased in *Atm*-/- murine osteoblasts; however, this reduction was restored by the introduction of PKC delta (PKCδ) from the outside [133]. In patients with CLL, inhibiting the *ATM* gene results in resistance to p53-induced cell death and a reduction in the efficacy of therapies that damage DNA. For this reason, the treatment of CLL with ATM insufficiency requires the utilization of other methods that do not rely on p53 protein. Researchers have investigated how the ATM-null CLL genotype affects cellular responses to oxidative stress in order to identify potential treatment strategies. Authors have found that treating ATM-null cells with pro-oxidants reduced the binding of NF-E2 p45-related factor-2 to antioxidant response elements, resulting in decreased expression of target genes compared to ATM-wild type CLL [134]. Moreover, cells lacking ATM in CLL exhibited decreased antioxidant levels and increased mitochondrial ROS. As a consequence, CLL with ATM null showed increased susceptibility to pro-oxidants in both laboratory and living organisms. Tumors with 11q deletion or TP53 mutations did not show a significantly higher sensitivity. The research conducted found that cell death was promoted by a mechanism independent of p53 or caspase and was associated with the activity of apoptosis inducing factor. Considering all these discoveries, it seems that targeting defective redox homeostasis might be a potential treatment approach for ATM-null CLL [134].

#### 2.1.3. S70pBcl2 

The Bcl-2 phosphorylation at serine-70, also known as S70pBcl2, offers protection against the death of cells that are caused by drugs. On the other hand, the precise role that it plays in the development of drug resistance is not yet fully known. Researchers have demonstrated that S70pBcl2 improves the survival of cancer cells by acting as a redox sensor and modulator to prevent DNA damage and cell death that are caused by oxidative stress. In primary cells taken from individuals suffering from CLL and lymphoma, as well as in cell lines that have been treated with ROS or chemotherapeutic medicines, there is a negative link between higher levels of S70pBcl2 and DNA damage. Through bioinformatic research, it was shown that S70pBcl2 is associated with a reduced median overall survival rate in lymphoma patients. Prolonged expression of the redox-sensitive S70pBcl2 has been shown to minimize DNA damage and cell death caused by oxidative stress by suppressing the generation of mitochondrial ROS. Investigators further show that S70pBcl2 reduces the binding of Bcl-2 to the mitochondrial complex-IV subunit-5A, leading to an impact on the function of mitochondrial complex-IV, respiration, and the production of ROS. Targeting S70pBcl2 with the phosphatase activator FTY720 is linked to heightened drug-induced DNA damage and cell death in primary CLL cells. Authors have shown that phosphorylation of serine-70 on the anti-apoptotic Bcl-2 acts as a redox sensor to protect against drug-induced oxidative stress-related DNA damage and cell death, potentially leading to therapeutic benefits [135]. 

#### 2.1.4. BACH2, PRDM1 

In a study, the expression of the BACH2 and PRDM1 genes was investigated in individuals with CLL who had not been treated with any drug that may potentially affect their immune response. It is interesting to note that the expression of BACH2 reduces in leukemic B cells from our CLL patients, although there were no alterations found in PRDM1 expression when compared with normal HDs of the same age. A deficit in BACH2 might result in checkpoint control signaling that is not properly controlled, which could then lead to B-cell precursor leukemia cells [136]. The findings of the study showed that the downregulation of BACH2 in normal lymphocytes leads to an increase in the resistance to apoptosis that is associated with aging. These changes were significantly more evident in T cells and B cells in individuals with CLL [137].

#### 2.1.5. p66Shc

Oxidative stress and apoptosis are linked, and the adaptor protein p66Shc is involved in the signaling pathways in this process [138]. P66Shc induces the generation of ROS by disrupting the mitochondrial respiratory chain through the binding and oxidation of cytochrome c. This finally leads to the initiation of the apoptotic cascade. In particular, P66Shc plays a crucial function in transmitting apoptotic responses to oxidative stress. This role was first identified in fibroblasts and has been found to be present in T lymphocytes as well [139,140]. In T lymphocytes, the presence of p66Shc leads to heightened vulnerability to apoptosis triggered by both pharmacological and physiological factors [141]. Within the CH2 domain, the phosphorylatable S36 residue was shown to be the location of the pro-apoptotic action of p66Shc [141]. The pro-apoptotic function of p66Shc is achieved by disrupting the integrity of mitochondria, leading to the loss of mitochondrial transmembrane potential and the release of cytochrome c [140]. This activity is dependent on the presence of both Lck and CamKII [142]. Another mechanism by which p66Shc promotes cell death is by influencing gene transcription, therefore tipping the balance of the BCL2 family’s pro- and anti-apoptotic members (BCL2L1, BAX) in favor of the pro-apoptotic ones. In addition, the production of p66Shc disrupts the balance of Ca^2+^ levels, leading to a negative impact on the survival of T cells. This effect is evident in T cells that overexpress p66Shc, as they demonstrate impaired capacity to handle excessive Ca^2+^ levels [140]. The pro-apoptotic function of p66Shc remains intact in B cells, as evidenced by its capacity to amplify B-cell apoptosis triggered by prolonged surface Ig cross-linking or by administration of the pro-apoptotic medication fludarabine [143]. Like T cells, this process entails a regulation of the expression of the BCL2 family proteins, resulting in a decrease in the levels of anti-apoptotic proteins (BCL2, BCL2L1) and an increase in the levels of pro-apoptotic proteins (BAX, BAK). The observations suggest that p66Shc may impair B-cell survival, which is supported by the fact that it reduces BCR-triggered, AKT-dependent survival signaling [143]. Additionally, P66Shc regulates the surface expression of CCR7, CXCR4, and S1PR1 during B-cell trafficking [144,145]. This control happens through transcriptional and posttranslational mechanisms. Furthermore, p66Shc can reduce the activity of the CXCR4 and CXCR5 signaling molecules [146]. There is a defect in the expression of p66Shc6 and its transcription factor STAT410 in CLL cells, which may be the reason of their extended longevity [143,144,147]. There appears to be a correlation between the absence of p66Shc and the onset of CLL. Upregulation of TCL1 in mice through the IgM heavy-chain enhancer (Eμ-TCL1) leads to the formation of a B-cell leukemia resembling aggressive CLL [148]. An investigation was conducted to examine the influence of p66Shc ablation on the severity and course of the disease in the Eμ-TCL1 mice model of chronic lymphocytic leukemia model. In this study, Patrussi et al. demonstrated that Eμ-TCL1/p66Shc-/- mice exhibited a more severe illness with an earlier start, greater incidence, and earlier mortality compared to Eμ-TCL1 animals. Eμ-TCL1/p66Shc-/- mice showed a significant increase of leukemic cells in both lymph nodes and other tissues. A correlation was found between the selectiveness of the target organ and the overexpression of receptors for chemokine, the ligands of which are expressed inside the target organ. In chronic lymphocytic leukemia cells, the level of p66Shc expression was observed to have an inverse correlation with chemokine receptor expression and the degree of organ infiltration. In Eμ-TCL1 mice, p66Shc expression decreased as the illness advanced but could be revived by administering the Bruton tyrosine kinase inhibitor ibrutinib. Their findings emphasize that p66Shc shortage plays a crucial role in the advancement and severity of CLL, highlighting p66Shc expression as a significant target for therapy [149]. An intriguing finding is that p66Shc can be regenerated in both CLL cells and leukemic Eμ-TCL1 cells with the use of ibrutinib, which also stimulates the expression of STAT4 in leukemic Eμ-TCL1 cells. Ibrutinib influences the expression of genes that are part of the BCR and CXCR4 pathways, which play a role in CLL. This indicates that STAT4 and its target p66Shc might be controlled through these mechanisms. The results emphasize the negative impact of p66Shc deficiency on CLL and emphasize the chemokine receptor network as a key focus of its effects [150]. 

#### 2.1.6. TSPO

The translocator protein, also known as TSPO, is a transmembrane protein with a molecular weight of 18 kilodaltons. It plays a crucial role in protecting the mitochondrial permeability transition pore, which is mostly situated in the outer mitochondrial membrane. There are a variety of biological mechanisms that are linked to TSPO. These activities include cell death, the control of cellular proliferation, porphyrin transportation and heme synthesis, immune system regulation, anion transport, and the control of the synthesis of steroids. Consequently, TSPO has been suggested as a potentially useful target for the development of new therapeutic drugs, notably for the treatment of cancer, by a number of studies. In the De Rosa et al. investigation, the response of 30 consecutive patients CLL to therapy with bendamustine and rituximab was examined according to the levels of TSPO expression. Furthermore, the concentrations of thiobarbituric acid reactive substances (TBARS) and NO, together with the enzymatic activity of caspase 3, were quantified. The lymphocytes of the 30 consecutive patients with CLL exhibited elevated levels of TSPO expression, decreased levels of TBARS and NO, and diminished caspase 3 activity in comparison to those of healthy individuals. After six months of therapy, the ratio of TSPO to mitochondria in 24 out of 30 patients with CLL approximated that of healthy individuals. Furthermore, there was an apparent elevation in TBARS and NO levels, which are two indicators of oxidative stress, as well as an enhancement in caspase 3 activity in all patients who responded to the treatment. Significantly, the six patients who exhibited resistance to therapy also demonstrated elevated TSPO levels, reduced caspase 3 activity, and decreased TBARS levels [151]. 

#### 2.1.7. Prognosis, Oxidative Stress and CLL

In recent decades, numerous prognostic factors have been studied in CLL, such as CD38 and ZAP-70 expression, as well as TP53 and the mutational status of the immunoglobulin heavy-chain variable region gene, which serves as independent prognostic factors and correlated with bad prognosis in CLL patients. In a publication, we reported that the elevated concentrations of carbonyl groups detected in CLL patients were positively related with CD38 expression and negatively linked to ZAP70 expression [152]. Furthermore, some correlations between OS status and polymorphisms of the Multi-Drug-Resistence-1 gene (i.e., involved in the protection against OS) were observed [153]. Moreover, a study evaluated the concentration of OS in subjects with CLL followed by an evaluation of its association with prognosis. Results demonstrated that oxidative stress correlated with abnormal immunophenotyping, cytogenetic changes, bad prognosis, Th9 cells, and overexpression of IL-9. Then, levels of IL-9 and Th9 cells were strongly correlated with oxidative stress and bad prognostic markers in CLL [154]. Finally, a photometric test and d-ROMs test were demonstrated to have important predictive significance. d-ROMs assay measures the total oxidant capacity of a serum/plasma sample against the N,N-diethylparaphenylenediamine that is employed as chromogenic substrate. A study reported a significant correlation between high d-ROMs values and the presence of an unmutated IgVH status, unfavorable cytogenetics, and a more advanced clinical stage [155,156]. Thus, OS status evaluation at diagnosis might have prognostic impact in CLL and could be helpful for a better interpretation of the biological mechanism of the disease.

## 3. Old and Potential New Therapies “ROS-Based” in CLL

### 3.1. The Role of Adaphostin

Certain studies have emphasized the significance of adaphostin, which was initially discovered as a suppressor of p210Bcr/abl kinase and a powerful catalyst of myeloid cell demise in p210Bcr/abl-positive K562 cells in laboratory conditions [157]. Adaphostin belongs to the Tyrphostins family, a group of small compounds specifically designed to function as inhibitors of tyrosine kinases [158]. Adaphostin causes various disruptions in cell signaling processes (Table 3), such as the secretion of cytochrome c and apoptosis inhibiting factor (AIF) in a dose-dependent manner, deactivation of protective pathways (Raf1, mitogen-activated protein kinase (MEK), extracellular signal-regulated kinase ½ (ERK1/2), and Akt), activation of stress-induced pathways (Jun N-terminal kinase (JNK) and p38 mitogen-activated protein kinase (MAPK)), and removal of phosphate groups from retinoblastoma protein (pRb) [159]. Subsequent research has shown that this substance may cause cell death via increasing reactive ROS levels [160] or reducing the expression of vascular endothelial growth factor (VEGF) [161,162] instead of by inhibiting p210Bcr/abl. Caspase inhibition proved ineffective in preventing adaphostin-induced mitochondrial damage or the majority of the observed changes in cell signaling. This finding aligns with prior observations indicating that inhibiting caspase did not affect the production of ROS [160]. In line with this hypothesis, adaphostin has the ability to trigger apoptosis in leukemia cells that lack the Bcr/abl mutation, such as CLL B [160]. In addition, a study assessed the capacity of adaphostin to trigger programmed cell death in CLL B cells and enhance the effects of fludarabine in laboratory conditions. Analyzing the annexin V/propidium iodide (PI) staining data showed that the amount of adaphostin needed to cause 50% cell death (IC50) within 24 h was 4.2 uM for CLL isolates, but it was more than 10 _M for B and T cells obtained from healthy donors. Immunoblots demonstrated that adaphostin induced the separation of poly(adenosine diphosphate-ribose) polymerase (PARP) and caspase-3 substrates, suggesting that adaphostin initiates apoptosis. Adaphostin elevated the concentration of ROS in CLL B cells, and the antioxidant N-acetylcysteine inhibited both the production of ROS caused by adaphostin and the process of death. Adaphostin also induced a reduction in the expression of the antiapoptotic protein Bcl-2. An enhanced apoptotic response was seen when adaphostin was administered in conjunction with fludarabine (F-ARA-AMP) [163]. Overall, the research indicates that adaphostin is toxic to primary CLL B cells, operates through a unique mechanism by generating ROS, and shows some preference for leukemic B cells. The drug has action against both low-risk and high-risk subtypes of CLL B cells, and it synergizes with fludarabine in vitro. This is an important property. Further preclinical assessment of adaphostin as a possible therapy for CLL is justified [163].

### 3.2. Role of ROS in Bendamustine Treatment

Bendamustine hydrochloride possesses a unique molecular structure that enables it to function both as an alkylating compound and a purine analogue. This medicine has demonstrated therapeutic efficacy in several malignancies, such as CLL, either as a standalone treatment or in conjunction with other medications [164,165,166,167,168]. Evidence has shown that bendamustine does not build up in the plasma of individuals who have been treated with it, indicating that it does not display any cross-resistance with commonly used alkylating medications [169]. Schwanen et al. reported bendamustine as having the capacity to induce cytotoxicity in most cell lines and primary tumor cells obtained from patients with CLL. This cytotoxicity is induced by both p53-dependent and p53-independent mechanisms, leading to the activation of several cell death effectors. The probability that bendamustine may bypass this major DNA damage sensor to carry out its function is very significant. The reason for this is that p53 mutations are frequently associated with the cause of CLL and MCL. The mitochondrial alteration hypothesized to occur in cells treated with bendamustine has not been clearly seen in malignant lymphoid B cells, despite its crucial role in the intrinsic cell death signaling pathway [170]. Moreover, studies have demonstrated that bendamustine induces cell death in CLL and MCL cells by activating the mitochondrial apoptotic pathway through the increased expression of the pro-apoptotic proteins PUMA and NOXA. These two proteins, belonging to the BCL-2 family, are crucial mediators of the cell reaction to stress in lymphoid diseases [171,172]. While the production of PUMA is believed to rely solely on p53’s ability to activate transcription [173], increasing evidence indicates that NOXA can be controlled by both p53-related and p53-independent systems [172,174,175]. Consistent with this, Roué et al. demonstrated that bendamustine therapy specifically increased the expression of PUMA in instances of CLL and MCL that have a functioning p53. This indicates the participation of this BH3-only protein, specifically in p53-dependent signaling. However, the activation of NOXA in cells treated with bendamustine is not limited to situations where p53 is wild type. In addition, the increase in expression might be noticed at lower doses of bendamustine in cells lacking functioning p53 compared to wild-type cells, a feature that has been previously reported in other experimental systems [172,176]. The authors’ observations indicate that the increase in NOXA and the activation of apoptotic signaling, which occurs independently of p53, following bendamustine therapy may be controlled by the amounts of ROS within the cell. This is supported by the fact that both of these processes may be significantly reversed by the ROS scavenger GSH [177]. On the other hand, for complete restoration of cell viability, GSH must be combined with the pan-caspase inhibitor z-VAD. fmk after it has been delivered. Hence, the generation of ROS could play a role in the caspase-independent mechanism that occurs simultaneously with the caspase-dependent pathway in cells subjected to bendamustine treatment. After being exposed to various input like tumor necrosis factor, proteasome inhibitors, or genotoxic medicines, it has been found that caspase-independent cell death is linked to oxidative stress and the production of ROS in several cancer models [172,178,179].

### 3.3. Auranofin

Auranofin (Ridaura) is a chemical that contains gold (I) and was granted approval by the United States Food and Drug Administration in 1985 as a main therapy for rheumatoid arthritis [180]. One of the key mechanisms of action that has been found for auranofin is that it acts as a pro-oxidant agent by interrupting the reduction and oxidation (redox) system that is present during cellular metabolism. The process works by blocking thioredoxin reductases (TrxRs), which are represented by two selenoenzyme isoforms: TrxR1 in the cytoplasm and TrxR2 in the mitochondria. This enzymes function as antioxidants by controlling the amounts of ROS, therefore safeguarding cells against the harmful effects of oxidative stress [181]. The essentiality of cytoplasmic Trx1 in its reduced form is crucial because of its multifaceted involvement in cellular activities, including the prevention of cell death by binding to apoptotic signaling kinase 1 (ASK1). Oxidation of Trx1 by ROS causes Trx1 to separate from the pro-apoptotic protein ASK1, hence initiating ASK1-induced cell death through the cytoplasmic c-JUN N-terminal kinase (JNK) and p38 MAP kinase pathways [45]. Trx1 additionally participates in cell growth and proliferation by suppressing phosphatase and tensin homolog (PTEN) and enhancing AKT action [182]. Ultimately, it was demonstrated that Trx1 relocates from the cytoplasm to the nucleus and stimulates several transcription factors, such as NF-kB and cancer suppressor p53 [183]. Considering the biological effects of Trx1, it is anticipated that the action of auranofin in inhibiting TrxR1 will result in elevated levels of ROS, stimulation of ASK-induced cell death, and hindered proliferation, development, and survival, owing to decreased AKT activity and transcription mediated by NF-kB and p53 [184]. Auranofin has shown effectiveness in preclinical studies using CLL cells. In both cultured and primary CLL cells, auranofin was able to elicit a deadly endoplasmic reticulum stress reaction. This was accomplished by blocking the activity of thioredoxin reductase and raising the amounts of ROS inside the cell. Auranofin showed enhanced lethality when used with heme oxygenase-1 and glutamate-cysteine ligase inhibitors against CLL cells. Auranofin successfully overcame opposition to cell death caused by protecting stromal cells and effectively eliminated primary CLL cells with chromosomal 11q or 17p deletions. Auranofin therapy significantly decreased tumor cell load and enhanced animal lifespan in TCL-1 transgenic mice, which serve as an in vivo model of CLL [185].

### 3.4. Acacetin 

The logic behind developing anticancer therapies based on mitochondrial targeting is well supported by research. The process of programmed cell death is triggered by the crucial occurrence of mitochondrial membrane permeabilization, regulated by the permeability transition pore complex known as the mitochondrial permeability transition (MPT) pore. This system is composed of multiple proteins and is located at the junction of the mitochondrial inner and outer membranes [186]. Tumor cells display different levels of mitochondrial disorders, including heightened transmembrane potential, increased ROS generation, and alterations regarding energy metabolic processes [187]. These changes offer an opportunity to selectively target cancer cell mitochondria to enhance therapeutic specificity [188]. Acacetin (5,7-dihydroxy-4′-methoxyflavone) constitutes a flavonoid chemical with anti-inflammatory, antiperoxidant, antiplasmodial, antimutagenic, and anticancer properties. Acacetin has been demonstrated to have an antiproliferative impact via promoting apoptosis and inhibiting progression of the cell cycle [189]. Research has shown that acacetin caused a dose-dependent increase in the generation of ROS in human osteosarcoma cells (HOS). Furthermore, in the same study, when the cells were pre-treated with the ROS inhibitor GSH, it greatly reversed the inhibitory effects of acacetin on cell growth and induction of apoptosis. The levels of caspase-3, -8, -9, Bax, and PARP were notably elevated, while the expression of Bcl-2 was reduced. This suggests that the generation of ROS can trigger DNA damage and result in the death of OS cells. Prior research has demonstrated that the JNK signaling pathway is capable of transmitting oxidative stress signals and triggering cell death in response to different stress stimuli. The authors of this study discovered that the phosphorylation of JNK and c-Jun was notably increased following the administration of acacetin. Through the use of the JNK inhibitor SP600125, it has been verified that JNK activation is connected to the control of acacetin-induced apoptosis [190]. A study examined the impact of acacetin (4′-methoxy-5,7-dihydroxyflavone) on CLL B lymphocytes and mitochondria in both laboratory and living organisms. CLL B cells were acquired from CLL individuals, whereas healthy B lymphocytes were collected from healthy donors. Mitochondria were extracted from B lymphocytes in both groups. Acacetin was tested for toxicity and anti-CLL activity using xenografts in severe combined immune deficient mice. In this study, the scientists investigated and examined the mechanism of action of acacetin on chronic CLL and healthy B cells, as well as their mitochondria. The authors showed that acacetin at an amount of 10 mM can trigger apoptosis in CLL B-lymphocytes by focusing on mitochondria directly. This process involves a spike in ROS, collapse of mitochondrial membrane potential (MMP), MPT, release of cytochrome c, activation of caspase 3, and ultimately apoptosis. Normal healthy B cells remain unaffected at similar concentrations. Additionally, administering acacetin orally demonstrated strong in vivo anticancer effects in CLL xenograft mice models. The data that they obtained in vivo suggest that acacetin accumulates and kills CLL B lymphocytes in a manner that is quite selective. The results suggest that acacetin selectively concentrates and eliminates CLL B lymphocytes by targeting malignant mitochondria and inducing ROS production, ultimately leading to CLL treatment. The data presented offer the justification and foundation for additional clinical assessment of acacetin to enhance patient treatment in CLL. These findings emphasize the role of ROS-mediated mitochondrial signaling in the specific effect of acacetin on CLL B lymphocytes [191].

### 3.5. Conus Textile 

Natural chemicals that originate from marine environments have been regarded by a significant number of researchers as one of the most important sources for the discovery of new drugs. Recently, these substances have been utilized in treating cancers because of their diverse pharmacological characteristics. Marine cone snails (Conus) are a widespread species of predatory predators found in tropical and subtropical waters. Cone snail venoms possess many medicinal benefits, including anti-cancer properties. Moreover, the venom of these sea creatures is abundant in a peptide known as conotoxins. Conotoxins have been documented to possess various pharmacologic and physiological effects [192,193,194]. A study conducted by Salimi et al. in 2020 demonstrated that crude venom of Conus textile has the capacity to generate ROS and induce apoptotic signaling in U87MG human glioma cells by way of the mitochondrial route [195]. The release of a cardiotoxin III-like peptide initiated apoptosis through the mitochondria-mediated pathway. This process involved an increased Bax/Bcl-2 ratio, the expulsion of cytochrome c, and the activation of caspase 9 [195]. Another study was conducted with the intention of determining whether or not crude venom of Conus textile and its fractions (A and B) had any apoptotic effects on CLL. On the basis of the findings, it was determined that the crude venom obtained from Conus cloth and its fraction B greatly decreased the viability of CLL B-lymphocytes. There was also a correlation between the exposure of CLL B-lymphocytes to fraction B and an increase in the amount of ROS, the collapse of the matrix metalloproteinase (MMP), disruption to the lysosomal membrane, and activation of caspase-It was determined that fraction B from crude venom of Conus textile exerts selective toxicity on CLL B lymphocytes, while having essentially little impact on normal lymphocytes. This conclusion was reached based on the aforementioned findings. In addition, this venom fraction has the potential to be an effective candidate for the induction of apoptosis in individuals who have CLL by means of the mitochondrial route [196].

### 3.6. Motexafin Gadolinium

An artificial expanded porphyrin known as motexafin gadolinium (MGd) selectively builds up in cancer cells and oxidizes a variety of intracellular metabolic products. These metabolites include ascorbate, nicotinamide adenine dinucleotide phosphate, glutathione, and protein thiols. This mechanism, which is referred to as futile redox cycling, results in the generation of ROS. Its use in hematologic malignancies is based on its ability to preferentially accumulate in tumor cells, which is similar to natural porphyrins. It operates through a distinctive process that induces oxidative stress and initiates apoptosis in several types of cancers. MGd triggers apoptosis in B lymphocyte NHL, CLL, and highly resistant myeloma cell lines. In addition, MGd has shown additive or synergistic effects when combined with ionizing radiation, a number of chemotherapeutic drugs, and rituximab in both in vitro and in vivo tumor models studies. Gene expression profiling shows an increase in stress-related genes in response to MGd, including as metallothioneins, heat shock proteins, and heme oxygenase. Initial findings from clinical trials involving MGd in hematopoietic malignancies indicate that it is well received, with minimal blood-related side effects. It has demonstrated effectiveness when used alone in CLL/SLL patients who have undergone extensive prior treatments. Additionally, when combined with 90Yttrium-ibritumomab, it has rapidly achieved full remission in relapsed NHL patients in the first two groups of participants. Ongoing clinical trials are investigating MGd as a standalone treatment and in conjunction with radiation and/or chemotherapy for hematologic malignancies [197].

### 3.7. Rigosertib 

A styryl benzylsulfone, rigosertib (ON 01910.Na), is an inhibitor of several kinases that does not compete with ATP and has strong anti-cancer and anti-mitotic effects. ON 01910.Na triggered programmed cell death in CLL B cells without causing notable harm to T cells or healthy B cells. ON 01910. The activity of sodium was comparable when it came to leukemic cells that were linked with a more severe illness course (IGHV unmutated, adverse cytogenetics) as opposed to cells that did not possess these characteristics. Gene expression analysis identified two primary modes of action: inhibiting PI3K/AKT and inducing ROS, leading to oxidative stress response by activating AP-1, c-Jun NH2-terminal kinase, and ATF3, ultimately resulting in the overexpression of NOXA. Cells were protected against drug-induced apoptosis by scavenging ROS and using short hairpin RNA (shRNA) to reduce the levels of ATF3 and NOXA [87]. The TME is now acknowledged as a major component in causing treatment resistance in CLL [198]. Despite the fact that treatment with a pan-PI3K inhibitor is sufficient to reverse the increase in CLL viability that was found in co-culture with stroma cells [199] or that was given by CD44 activation [200], it does not induce substantial apoptosis on its own. Conversely, ON 01910.Na not only inhibited the pro-survival impact of follicular dendritic cells but also triggered apoptosis in over 50% of cells, even in the presence of stroma. All of these factors indicate the possibility that the combination of a PI3K inhibitor with an agent that induces reactive oxygen species (ROS) might potentially give additive anti-tumor actions [87].

### 3.8. Arsenic Trioxide

The use of arsenic trioxide as an effective treatment for acute promyelocytic leukemia has seen a resurgence in favor within the mainstream medical community over the course of the past 15 years [201]. In the context of this particular condition, arsenic trioxide induces differentiation in this circumstance by degrading the PML-RARα fusion protein. Arsenic trioxide also can induce apoptosis by several mechanisms such as mitochondrial damage, cell cycle arrest, and the generation of superoxide radicals in a caspase-dependent or -independent manner, depending on the cell type [202,203,204,205,206,207]. Myeloma tumor cells without p53 are specifically targeted and eliminated by As2O3, triggering an extrinsic apoptotic pathway instead of an intrinsic one [208]. An investigation showed that arsenic trioxide can cause B-CLL cells to die, and that it kills cancerous B cells more effectively than healthy ones, affecting mainly CLL cells from high-risk patients (del17p13, high CD38 expression or unmutated IgVH status). The cellular death caused by arsenic trioxide is often characterized as conventional apoptosis. Authors have suggested that the susceptibility of BCLL cells to arsenic trioxide is determined by the interaction of arsenic trioxide with enzymes from the redox system, since the apoptosis generated by arsenic trioxide can be fully prevented by antioxidants [209]. When B-CLL cells are subjected to in vitro treatment with arsenic trioxide, the overexpression of many mRNAs that code for antioxidative enzymes occurs. These enzymes include glutathione reductase, thioredoxin reductase, and superoxide dismutase 1, among others. Other factors contributing to the increased SOD activity in high-risk individuals are also possible. Elevated levels of interleukin-1 and -6 in the plasma of B-CLL patients are linked to decreased 3-year survival and have been demonstrated to trigger SOD2 [210,211]. To summarize, arsenic trioxide shows good selectivity for tumor cells and strong efficacy, especially in cells from high-risk B-CLL patients, making it a potential treatment for this specific patient subgroup. More in vitro research is required in order to assess the potential utility of arsenic trioxide in conjunction with other cytotoxic medications and in chemoimmune treatment regimens that are already being utilized for these patients. This will allow for the determination of its optimal administration, either in combination or in sequential regimens [209]. Additionally, arsenic trioxide increases Heme oxygenase-1 (HMOX1) synthesis in several cell systems, mostly through ROS-induced signaling pathways [212,213,214,215]. HMOX1 is the primary antioxidant enzyme in cells, playing an essential function in eliminating intracellular ROS and regulating several biological functions such as cell survival, growth, and inflammation. Several studies in the literature provide evidence for the anti-apoptotic and protective function of HMOX1 in different injury models [216,217]. Specifically, Amigo-Jiménez et al. investigated the gene expression profile that is triggered by arsenic trioxide in CLL tumor cells in order to obtain deeper insight into this topic. Arsenic trioxide influenced several genes, particularly those related to oxidative stress, with HMOX1 being the most significantly elevated gene, also showing increased expression at the protein level. Arsenic trioxide also elevated MMP-9 at both the mRNA and protein levels, as previously reported. The investigators showed that the increase of MMP-9 by arsenic trioxide is linked to the activation of the p38 MAPK/AP-1 signaling pathway by the use of particular inhibitors, qPCR studies, and gene silencing techniques. Additionally, suppressing HMOX1 gene or blocking HMOX1 function increased p38 MAPK phosphorylation and c-jun expression/activation, leading to the transcriptional upregulation of MMP-9. Increased expression of HMOX1 or augmentation of its function resulted in an adverse effect. Cell survival tests showed that altering HMOX1 expression or activity revealed HMOX1’s pro-apoptotic function and increased the toxic impact of arsenic trioxide on CLL cells. Their findings shed light on a previously unknown pathway by which HMOX1 regulates MMP-9, an anti-apoptotic protein, and the CLL cells response to arsenic trioxide. HMOX1 emerges as a novel therapeutic target in CLL, and the use of HMOX1 modulators in conjunction with arsenic trioxide might be an effective treatment approach in CLL [218].

**Table 3:** Overview of the biological mechanism/target of ROS-based agents.

Drugs/Agents	Biological Mechanisms/Target
Adaphostin	Disruptions in cell signaling processes, such as the secretion of cytochrome c and apoptosis inhibiting factor (AIF) in a dose-dependent manner, deactivation of protective pathways (Raf1, MEK, extracellular ERK1/2 and Akt), activation of stress-induced pathways (JNK and MAPK), and removal of phosphate groups from RB [159]
Bendamustine	Cytotoxicity p53-dependent and p53-independent mechanism; increased expression of the pro-apoptotic proteins PUMA and NOXA [177]
Auranofin	Inhibit TrxRs [181]
Acacetin	Phosphorylation of JNK and c-Jun [190]
Conux Texile	Release of a cardiotoxin III-like peptide [195]
Motexafin gadolinium	Futile redox cycling [197]
Rigosertib	Inhibit PI3K/AKT and induce ROS, leading to oxidative stress response by activating AP-1, c-Jun NH2-terminal kinase, and ATF3, ultimately resulting in the overexpression of NOXA [87]
Arsenic trioxide	Degrade the PML-RARα fusion protein [201] Increase HMOX1 [213]

## 4. New Perspectives: Nanotechnology 

Cancer therapy is being confronted with several obstacles, including medication resistance and systemic toxicity. Nano-systems, an advanced use of nanotechnology, have transformed cancer treatment by enhancing the targeting of ROS in the Tumor Microenvironment (TME). Physicist Robert Feynman initially proposed the notion of nanotechnology in 1959 at a lecture at Caltech University [219]. These sophisticated technologies improve the accuracy of therapeutic administration, reducing harm to healthy cells and boosting treatment specificity. Nano-systems have the advantage of efficiently delivering a large quantity of a substance to a specific location, enhancing its stability, and prolonging its circulation in the blood while reducing potential side effects, such as those related to the systemic effects of chemotherapy drugs [220]. In addition, nanotechnologies make it possible to encapsulate and distribute hydrophobic compounds, which are often difficult to freely administrate, therefore enhancing the soluble nature and biocompatibility of these molecules. This is dependent on the composition of the system [221]. Nano-systems must be made up of materials that are non-toxic, biocompatible, and biodegradable in order for them to be suitable for use in environments that involve living organisms [222]. Nano-systems have been developed as a new method to trigger apoptosis in cancer cells by directly adjusting ROS levels, either raising or decreasing them [223,224,225]. The dimension and charge of nanomaterials, among their other physicochemical properties, make them extremely penetrating, making them ideal for targeting tumors. By targeting ROS metabolic pathways, ROS-based nanoparticles increase ROS production inside cells, leading to cancer cell death. The production of ROS nanoparticles can be induced in a number of different methods, including through the use of sonodynamic therapy, photodynamic therapy, chemodynamic therapy, and substances that are chemically delivered [226]. Some research emphasizes the potential of nanotechnology as a treatment approach for malignant hematological illnesses as CLL. As an illustration of the point made, it has been demonstrated via both preclinical and clinical research that G3139 (antisense to bcl2 mRNA’s first six codons) has the ability to dramatically induce apoptosis and diminish chemosensitivity in a variety of malignancies, including CLL [227]. The therapeutic advantage of targeting the delivery of G3139 with immunoliposome conjugated rituximab was validated by a trial that was conducted recently [228]. A different investigation was conducted by Chiang and colleagues, in which they created immunoliposomes loaded with miR-29b and then coupled this system with an antibody that targets the tyrosine-protein kinase transmembrane receptor (ROR1). Considering that ROR1 is mostly produced by leukemic cells that are associated with CLL, employing this strategy appears to be an effective method for selective targeting [229]. To summarize, research conducted on the use of nanomedicine-based therapy in the treatment of CLL has demonstrated the effectiveness of the method by reducing the severity of adverse effects and increasing the effectiveness of the therapy. Another example regards novel nanostructures known as gold clusters. These nanomaterials have a perfect atomic composition and are incredibly small in size. They also display special physicochemical and biological properties [230,231]. It was revealed that gold clusters that were coated with peptides or proteins have exceptional biocompatibility, which allows them to be acceptable for potential applications in the field of biomedicine [230,232]. The peptide-capped gold cluster (Au25Sv9) selectively targets abnormal oxidative stress in CLL cells to specifically block thioredoxin reductase (TrxR) activity, leading to substantial apoptosis. Yet, the large concentrations of the gold cluster needed to trigger apoptosis limit its potential for other uses. The latest research indicates that CLL cells have high levels of antiapoptotic BCL-2 protein to resist chemotherapy-induced cell death, suggesting that BCL-2 might be a potential target for CLL treatment. Unfortunately, the Au25Sv9 compound, which is not specific to mitochondria, has minimal impact on BCL-2. Authors in an investigation identified a modified BADBH3 peptide (B1P) that effectively blocked the BCL-2 protein in CLL cells. They discovered that B1P was able to efficiently enhance the sensitivity of MEC-1 cells to a low dosage of Au25Sv9. Researchers simplified the therapy schedule by creating a gold cluster coated with B1P peptides by one-step synthesis to include BCL-2 antagonistic activity, resulting in the formation of BGC. Low concentrations of BGC were observed to elicit greater apoptosis in MEC-1 cells compared to similar doses of the Au25Sv9 cluster or B1P peptide alone. Gold clusters reduced TrxR function and enhanced the activation of the BCL-2 family-mediated mitochondrial death cascade by binding to BCL-2 on mitochondria. These findings show that targeting the increased expression of BCL-2 in CLL cells, together with blocking TrxR at the same time using a single gold cluster, is a hopeful approach for treating CLL cells [233]. In conclusion, the future field of cancer therapy has great promise for improvements in nanotechnology. Ongoing studies and development will probably result in the incorporation of intelligent nano-systems, allowing for real-time monitoring and adaptive treatment approaches. Personalized medicine strategies will be crucial in customizing treatments for individual patients according to their specific TME traits. Furthermore, integrating nano-systems with other therapeutic methods like immunotherapy or gene therapy shows potential for enhanced effects and better results. Combining immunotherapy with nano-systems has the potential to enhance therapeutic results [223,224,225].

### New Prospectives: PROTACs

The development of protein-degrading complexes as potential therapies for a wide variety of disorders has seen a number of significant developments over the course of the past several decades. One of the most thrilling advancements in this field is the development of PROTACs, also known as Proteolysis Targeting Chimeras. PROTACs are tiny compounds that have two different functional groups and are capable of inducing the degradation of the Protein of Interest (POI) [234]. PROTACs consist of a weapon that is specifically aimed at the target of interest. This warhead is connected to a ubiquitin protein ligase (E3) ligase-recruiting element by a flexible linker. This enables the creation of a ternary complex (TC) consisting of the target, PROTAC, and an E3, leading to the destruction of the target through the proteasome. A series of enzymes known as ubiquitin-activating enzyme (E1), ubiquitin-conjugating enzyme (E2), and E3 are responsible for acting as catalysts in the process of polyubiquitylation of the target protein [235]. PROTACs often focus on E3 ligases such as cereblon (CRBN), murine double-minute 2 (MDM2), Von Hippel-Landau (VHL), and inhibitor of apoptosis protein (IAP) [236]. PROTACs offer a potential solution to address the limitations of existing cancer treatments, including drug resistance, the inability to target proteins without active sites, and the degradation of nonenzymatic protein functions by breaking down the entire molecule [237]. In order to target oncoproteins that are considered to be critical in the advancement of various types of leukemia, a number of PROTACs are currently being developed [238,239,240]. Regarding CLL, BTK has a role in the milieu surrounding the cell, where it plays a role in assisting cancer cells in their development and survival [241,242]. Multiple BTK-targeted PROTACs have been documented, demonstrating how PROTACs can effectively overcome the clinical limitations of ibrutinib. These findings showcase enhanced specificity and sustained efficacy in models of ibrutinib resistance [243]. Notably, PROTACs that aim against BTK often employ CRBN instead of VHL since VHL has inadequate degrading capabilities [244,245]. Specific BTK PROTACs were developed to selectively target the C418S mutation in BTK. Among these, MT-802 demonstrated complete degradation of BTK in CLL cells after approximately 4 h at a dose of 250 nM [245]. Furthermore, research was conducted to evaluate the efficacy of reversible covalent BTK-degrading PROTACs with their irreversible covalent and noncovalent equivalents. The findings demonstrated that although the noncovalent PROTACs were the most efficient, the reversible PROTAC (RC-3) nevertheless demonstrated great potency and selectivity, which led to a reduction in the number of reactions that were not intended to be produced [246]. PROTACs are a promising and viable approach to hinder BTK activity in CLL, serving as a valuable alternative therapy [247,248]. Specifically, many medicines have demonstrated the ability to maintain effectiveness against the Cys481Ser mutant BTK, which is a primary factor in the development of resistance to the drug ibrutinib. Furthermore, BTK-targeted PROTACs have a higher level of selectivity compared to ibrutinib, even when employing warheads that are very similar to those used by ibrutinib. Improved selectivity in this context refers to the ability to specifically target BTK through non-covalent binding. This selectivity may be due to stabilizing contacts between BTK and E3 ligase within the TC. Multiple studies have indicated an increased level of specificity for PROTACs [236,249,250]. Assessing the correlation between enhanced selectivity and decreased toxicity would necessitate the examination of suitable in vivo models, specifically focusing on the most severe toxicities associated with ibrutinib, such as atrial fibrillation and hemorrhage [243].

## 5. Discussion and Conclusions

The complex correlation between CLL and oxidative stress emphasizes the varied nature of this illness and brings attention to prospective treatment opportunities. CLL cells have increased amounts of ROS and oxidative stress as a result of disturbances in the redox equilibrium. This imbalance affects several cellular functions, including as protein function, transcription factor activity, and mitochondrial function, eventually affecting the survival and growth of cells. The dysregulation of genes involved in maintaining the balance of oxidative and antioxidative processes, including ATM, Bcl-2, BACH2, PRDM1, p66Shc, and TSPO, has a role in the development of CLL and its resistance to treatment. From a prognostic perspective, assessing oxidative stress levels at the time of diagnosis shows potential as a prediction technique in CLL. Biomarkers indicating oxidative stress are associated with the severity of the disease, anomalies in immune cell characteristics, and unfavorable genetic profiles. These biomarkers offer important information about the prognosis of the disease and help in making treatment decisions. Adaphostin, bendamustine, auranofin, acacetin, conus textile venom, motexafin gadolinium, rigosertib, and arsenic trioxide are a variety of substances that use ROS signaling to trigger cell death and prevent the growth of CLL cells. Additional investigation is necessary to obtain a deeper comprehension of the processes by which these therapies work and to enhance their utilization, either as standalone treatments or in conjunction with current medications. It is necessary to conduct clinical trials to assess the safety and effectiveness of these substances in patients with CLL, especially those with a high risk of the disease. These trials will help confirm the therapeutic benefits of these drugs. Nanotechnology and PROTACs provide potential opportunities for enhancing cancer treatment, namely in the realm of CLL. Nanotechnology enables an accurate localization of TME via nano-systems, hence reducing overall toxicity and improving the effectiveness of treatment. Researchers are using ROS-based nanoparticles and other nanomaterials, such as gold clusters, to investigate new methods of triggering apoptosis in cancer cells without harming healthy organs. In addition, the combination of nanotechnology with other therapeutic approaches like immunotherapy and gene therapy has the potential to provide synergistic effects, leading to more efficient and customized therapies. However, PROTACs are an innovative approach to specifically break down proteins, which has advantages over traditional treatments since it can overcome drug resistance and degrade proteins that are crucial for the advancement of CLL, such as BTK. PROTACs can overcome limitations of current therapies and provide improved selectivity and effectiveness using small compounds that engage E3 ubiquitin ligases to break down targeted proteins. Overall, nanotechnology and PROTACs show great potential in the field of cancer therapy, particularly in the treatment of CLL and other types of cancer. Nanotechnology facilitates accurate localization of cancer cells while avoiding unintended impacts, whereas PROTACs provide a new method to specifically break down disease-causing proteins. The incorporation of these groundbreaking technologies into current therapeutic approaches shows significant potential for enhancing patient results and propelling tailored cancer treatment methods. Additional investigation and clinical advancement are necessary to completely exploit the therapeutic capabilities of nanotechnology and PROTACs in the field of cancer. To summarize, comprehending the complex interaction between CLL and oxidative stress provides new understanding of how the illness develops and reveals possible targets for treatment. Targeting pathways related to oxidative stress may offer a viable strategy to enhance treatment results and prognosis in CLL. 

## Figures and Tables

**Table 1:** Overview of the principle recurrent gene mutations in CLL [12].

Types of Recurrent Gene Alterations	Genes
Signaling pathways of B-cell receptor and Toll-like receptor	*PAX5*, *MYD88*, *BCOR*, *IKZF3*
Splicing of RNA and metabolic processes	*U1*, *DDX3X*, *RPS15*, *SF3B1*
Cell cycle regulators	*CDKN1B*, *CDKN2A*
MAPK-ERK	*BRAF*, *NRAS*, *KRAS*
Changes in chromatin structure	*SETD2*, *KMT2D*, *CHD2*, *ASXL1*
NF-KB pathway	*TRAF2*, *TRAF3*, *BIRC3*

**Table 2:** Criteria for initiating treatment: “active disease” [1].

Criteria for Initiating Treatment: “Active Disease”
Indications indicate gradual decline in bone marrow function, characterized by the occurrence or deterioration of anemia and/or thrombocytopenia. When the amount of Hb is less than 10 g/dL or the platelet count is less than 100 × 10^9^/L, it is typically seen as an indication for therapy. Nevertheless, in certain individuals, platelet counts below 100 × 10^9^/L might persist without significant changes for an extended duration, and in such cases, there is no immediate need for therapeutic action.
Splenomegaly that is either large (≥6 cm below the left costal border), progressing, or causing symptoms.
Lymph nodes that are large (≥10 cm in longest diameter) or lymphadenopathy that is worsening or causing symptoms.
Increasing lymphocytosis is characterized by a significant rise of at least 50% during a period of two months, or a lymphocyte doubling time (LDT) of less than six months. The LDT may be calculated using linear regression extrapolation of absolute lymphocyte counts recorded every 2 weeks during a 2–3 month observation period. However, patients with baseline blood lymphocyte counts below 30 × 10^9^/L may need a longer observation time to accurately establish the LDT. When diagnosing lymphocytosis, it is important to rule out factors other than CLL, such as infections or the use of steroids.
Anemia and thrombocytopenia are examples of autoimmune complications that exhibit a diminished response to corticosteroids.
Extranodal involvement, whether symptomatic or functional, can occur in several parts of the body such as the skin, kidney, lung, and spine.
Signs and symptoms associated with the disease, as defined by any of the following sources: Unintentional weight loss of at least 10% within the preceding six months, significant fatigue (defined as ECOG performance scale 2 or worse; inability to work or execute typical activities), fevers of at least 100.5 °F or 38.0 °C for a period of two weeks or longer without any indication of infection, and night sweats for a period of at least one month without any indication of infection are all indicators of a possible infection.
